# The Correlations between the Intensity of Histopathological Ubiquitin-Specific Protease 11 Staining and Progression of Prostate Cancer

**DOI:** 10.3390/ph16121703

**Published:** 2023-12-08

**Authors:** Jae Heon Kim, Hee Jo Yang, Kwang Woo Lee, Jae Joon Park, Chang-Ho Lee, Youn Soo Jeon, Jae Ho Kim, Suyeon Park, Su Jung Song, Ji-Hye Lee, Ahrim Moon, Yon Hee Kim, Yun Seob Song

**Affiliations:** 1 Department of Urology, School of Medicine, Soonchunhyang University, Seoul 04404, Republic of Korea; piacekjh@schmc.ac.kr (J.H.K.); 88joony@naver.com (J.J.P.); 2 Department of Urology, School of Medicine, Soonchunhyang University, Cheonan 31151, Republic of Korea; 3 Department of Urology, School of Medicine, Soonchunhyang University, Bucheon 14584, Republic of Korea; 4 Department of Urology, School of Medicine, Soonchunhyang University, Gumi 39371, Republic of Korea; uroseven@schmc.ac.kr; 5 Department of Data Innovation, Soonchunhyang University Seoul Hospital, Seoul 04404, Republic of Korea; 6 Department of Applied Statistics, Chung-Ang University, Seoul 06974, Republic of Korea; 7 Soonchunhyang Institute of Medi-Bio Science, Soonchunhyang University, Cheonan 31151, Republic of Korea; ssong1@sch.ac.kr; 8 Department of Integrated Biomedical Science, Soonchunhyang University, Cheonan 31151, Republic of Korea; 9 Department of Pathology, School of Medicine, Soonchunhyang University, Cheonan 31151, Republic of Korea; whui01@schmc.ac.kr; 10 Department of Pathology, School of Medicine, Soonchunhyang University, Bucheon 14584, Republic of Korea; 11 Department of Pathology, School of Medicine, Soonchunhyang University, Seoul 04404, Republic of Korea

**Keywords:** ubiquitin, USP11, prognosis, prostate cancer

## Abstract

Background: Ubiquitin-specific protease 11 (USP11), one of the principal phosphatase and tensin homolog (PTEN) deubiquitinases, can reserve PTEN polyubiquitination to maintain PTEN protein integrity and inhibit PI3K/AKT pathway activation. The aim of the current study was to investigate the associations between immunohistochemical USP11 staining intensities and prognostic indicators in individuals with prostate cancer. Methods: Tissue microarrays (TMAs) were performed for human prostate cancer and normal tissue (control) samples. Data on patient’s age, Gleason score, plasma prostate-specific antigen (PSA) titer, disease stage, and presence of seminal vesicles, lymph nodes, and surgical margin involvement were collected. A pathologist who was blinded to the clinical outcome data scored the TMA for USP11 staining intensity as either positive or negative. Results: Cancerous tissues exhibited lower USP11 staining intensity, whereas the neighboring benign peri-tumoral tissues showed higher USP11 staining intensity. The degree of USP11 staining intensity was lower in patients with a higher PSA titer, higher Gleason score, or more advanced disease stage. Patients who showed positive USP11 staining were more likely to have more optimal clinical and biochemical recurrence-free survival statistics. Conclusions: USP11 staining intensity in patients with prostate cancer is negatively associated with several prognostic factors such as an elevated PSA titer and a high Gleason score. It also reflects both biochemical and clinical recurrence-free survival in such patients. Thus, USP11 staining is a valuable prognostic factor in patients with prostate cancer.

## 1. Introduction

Phosphatase and tensin homolog (PTEN) is a lipid phosphatase, which is an antagonist of class I phophatidylinositol 3-kinase (PI3K) signaling in the PI3K/AKT cascade. It is a well-established key dose-dependent cancer suppressor [1,2,3,4]. In the absence of PTEN, there is marked PI3K/AKT pathway amplification that can promote cellular growth, replication, migration, viability, and metabolism by phosphorylating its downstream signaling protein [3]. The downregulation of PTEN relies on the ubiquitin–proteasome system [5,6,7,8]. The polyubiquitination and breakdown of PTEN are facilitated by E3 ubiquitin ligases such as NEDD4-1 (neural precursor cell expressed developmentally downregulated protein 4-1), XIAP (X-Linked Inhibitor of Apoptosis), WWP2 (WW Domain Containing E3 Ubiquitin Protein Ligase 2), and CHIP (clonal hematopoiesis of indeterminate potential) [9,10,11,12]. De novo-identified enzymes including HAUSP (Herpesvirus-associated ubiquitin-specific protease), ataxin-3, ubiquitin-specific protease 11 (USP11), and OTUD3 (ovarian tumor-associated protease domain-containing protein 3) can lead to the deubiquitination of PTEN. HAUSP can selectively eradicate PTEN mono-ubiquitination to facilitate its transfer out of the nucleus [13]. Ataxin-3 controls PTEN at the point of transcription [14]. OTUD3, found in the cytoplasm, can influence the integrity of PTEN within the cytosol, specifically with respect to breast malignancy [15,16]. USP11, also referred to as UHX1 (ubiquitin hydrolase on the X chromosome), was first recognized in association with X-linked retinal disorder-related genes at the Xp11.23 locus [17].

Previous studies have demonstrated a frequently arising deletion of USP11 interval in ovarian malignancies [9]. The clinical remit of USP11 has been demonstrated to be significant following the analysis of TCGA data sets obtained from cBioPortal (www.cbioportal.org) and Kmplot (http://kmplot.com/analysis/, accessed on 23 October 2022) [9,10,11,12,18,19,20]. USP11 as an enzyme has its own unique features. It is responsible for the intranuclear depolyubiquitination and stabilization of PTEN. Consequently, it is responsible for PI3K/AKT stimulation. USP11 is an antagonist of PI3K due to its ability to amplify PTEN expression [5,6,7,17,21,22].

The purpose of the current study was to evaluate the relationships between the intensity of USP11 staining on immunohistochemical analysis and disease progression factors in individuals with prostate cancer.

## 2. Results

### 2.1. Clinicopathologic Properties of Patients with Prostate Cancer

**Figure 1:**
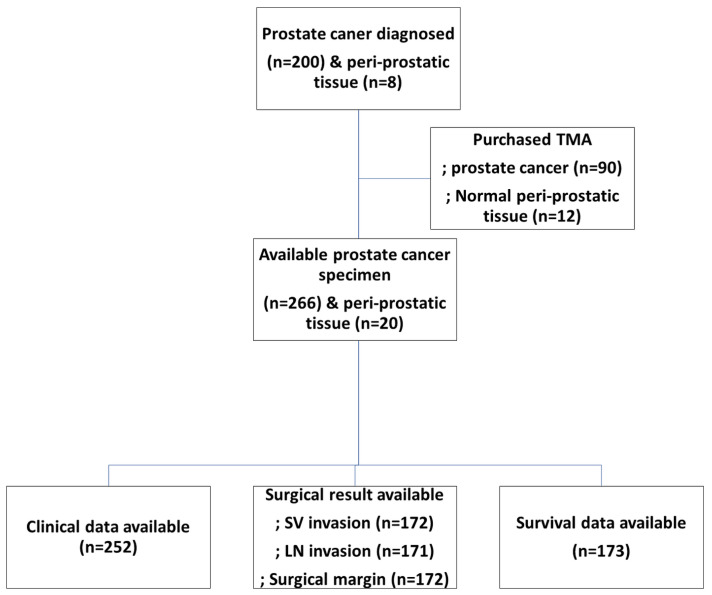
Number of available specimens for each test item.

A total of 286 tissue microarrays (TMA) were constructed. Among them, 20 RMAs were for adjacent non-neoplastic tissues around prostate cancer and 266 TMAs were for prostate cancer tissue (Table 1). Of these, the analysis was conducted in patients with prostate cancer (*n* = 266), excluding 24 patients who had inadequate patient data. The clinical data of 252 patients could be analyzed. Surgical outcomes, such as surgical margin involvement, could be analyzed in 172 patients from our hospital. Survival data were available for 173 patients (Figure 1).

### 2.2. USP11 Expression

**Figure 2:**
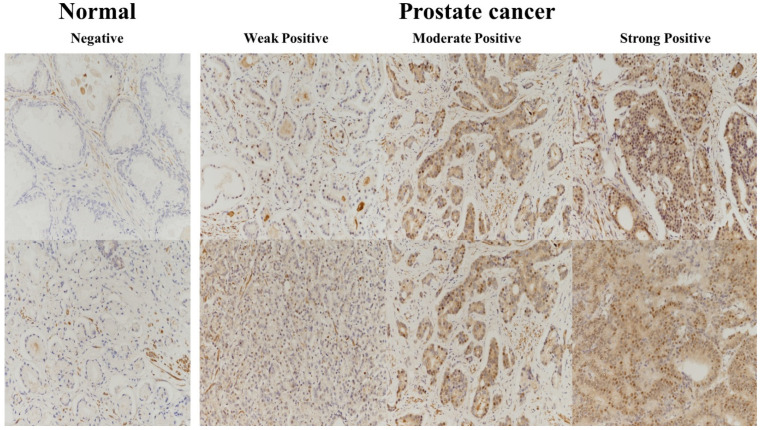
Representative photographs of USP11 expression in prostate cancer tissue and no USP11 expression in non-cancerous prostate tissue. The expression and location of USP11 were re-classified into the following two grades: negative (negative) and positive (weak, moderate, and strong). Original magnification: ×200.

Regarding UPS11 expression and staining score, 135 cases had a score of 0, and 131 cases had a score of 1. Non-neoplastic prostate tissue displayed a higher grade, whereas prostate cancer displayed a lower grade of USP11 (Table 2, Figure 2) (*p* < 0.001).

### 2.3. Association of USP11 Expression with Clinicopathologic Parameters through a Cox Proportional Hazard Model Test

#### 2.3.1. Univariable Analysis

The biochemical recurrence-free survival of prostate cancer patients with a lower grade of USP11 had higher prostate-specific antigen (PSA) titer than those with a higher grade of USP11 (*p* < 0.001). Patients with a lower grade of USP11 had higher Gleason scores than those with a higher grade of USP11 (*p* < 0.001) (Table 3). Patients with a lower grade of USP11 had a higher pathologic stage than those with a higher grade of USP11 (*p* < 0.001). Patients with a lower grade of USP11 had higher involvement of seminal vesicles than those with a higher grade of USP11 (*p* < 0.001). Patients with a lower grade of USP11 had a higher rate of positive surgical margins than those with a higher grade of USP11 (*p* < 0.001). Patients with a lower grade of USP11 had a higher incidence of lymph node involvement than those with a higher grade of USP11 (*p* < 0.001).

The clinical recurrence-free survival of prostate cancer patients with a lower grade of USP11 showed a higher PSA than those with a higher grade of USP11 (*p* < 0.05) (Table 4). Patients with a lower grade of USP11 showed higher involvement of seminal vesicles than those with a higher grade of USP11 (*p* < 0.05). Patients with a lower grade of UPS11 showed higher lymph node involvement than those with a higher grade of USP11 (*p* < 0.001). The overall survival of prostate cancer patients with a lower grade of USP11 showed higher involvement of seminal vesicles than those with a higher grade of USP11 (*p* < 0.05) (Table 5).

#### 2.3.2. Multivariable Analysis

Biochemical recurrence-free survival of prostate cancer patients with a lower grade of USP11 showed higher PSA titers than those with a higher grade of USP11 (*p* < 0.05). Patients with a lower grade of USP11 showed higher positive surgical margins than those with a higher grade of USP11 (*p* < 0.05). Patients with a lower grade of UPS11 showed higher lymph node involvement than those with a higher grade of USP11 (*p* < 0.05) (Table 6).

The clinical recurrence-free survival of prostate cancer patients with a lower grade of USP11 showed higher lymph node involvement than those with a higher grade of USP11 (*p* < 0.001). The overall survival of prostate cancer patients with a lower grade of USP11 showed higher involvement of seminal vesicles than those with a higher grade of USP11 (*p* < 0.05) (Table 7).

#### 2.3.3. Survival Analysis

**Figure 3:**
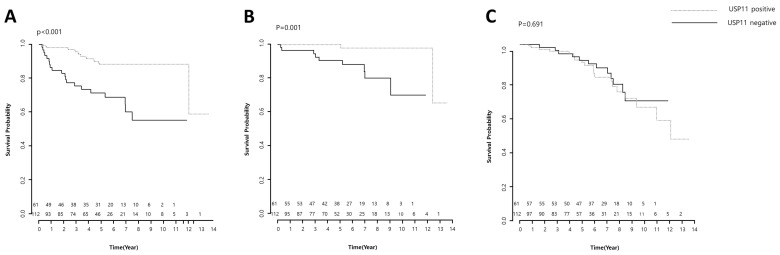
Kaplan–Meier survival analysis for USP11 expression in prostate cancer patients. (**A**,**B**) Prostate cancer patients with higher USP11 expression showed a better prognosis of biochemical recurrence-free survival and clinical recurrence-free survival than prostate cancer patients with lower USP11 expression (*p* < 0.001). (**C**) Prostate cancer patients with higher USP11 expression showed no better prognosis of overall survival than those with lower USP11 expression (*p* > 0.05).

Kaplan–Meier survival analysis for USP11 expression in prostate cancer patients showed that USP11 positivity was correlated with the biochemical recurrence-free survival of prostate cancer (*p* < 0.001) and clinical recurrence-free survival of prostate cancer (*p* < 0.001). However, USP11 positivity was not correlated with the overall survival of prostate cancer (*p* > 0.05) (Figure 3).

## 3. Discussion

In comparison with samples derived from prostate tumors, those obtained from neighboring benign tissues had higher levels of USP11 (*p* < 0.04) (Table 2; Figure 2). These results indicated that the expression of USP11 could facilitate differentiation of cancerous prostate cells from normal tissues. Previous studies using immunohistochemical staining have demonstrated a disparity in USP11 staining between cancerous prostate cells and normal cells. Data obtained from this study substantiate the importance of the deubiquitination process in tumorigenesis within the prostate gland and abnormal replication of malignant cells.

The downregulation of USP11 in human malignancies is positively associated with PTEN. A microarray database, which was published earlier, has demonstrated the expression of USP11 in human prostate and breast neoplasms. Studies employing transcriptome-profiling techniques to analyze prostate cancer in humans have shown a downregulated expression of USP11 transcription in primary prostate cancers, and they have also shown that such downregulation is strongly correlated with the aggressiveness of the malignancy [23,24]. The downregulation of USP11 is more extensive in prostate cancers that have spread remotely than in primary prostate cancer, with normal prostatic tissues showing the lowest degree of USP11 suppression [23]. Human breast cancers are also associated with diminished USP11 expression [25]. Cancer patient survival analyses have indicated the clinical relevance of downregulated USP11 expression, with individuals having higher USP11 expression experiencing a more favorable clinical outcome [26].

A lack of USP11 can lead to cell growth and migration and promote metabolism. In transgenic adenocarcinoma of the mouse prostate (TRAMP) mice, the advancement of malignancy is induced following the knockout of USP11 [27]. USP11 has been proposed to be a likely suppressive mediator for malignancies in relation to the onset of prostate cancer, its development, and remote dissemination. This action is dependent on PTEN, which is governed by cellular density. When levels of USP11 are low, PTEN amplification arising from high cell density is suppressed, implying that the latter governs the physiology of PTEN protein titers to some extent via USP11 transcriptional alteration [27].

Surprisingly, PTEN controls its own integrity via the transcriptional amplification of USP11 through the PI3K/Forkhead box transcription factors (FOXO) pathway. This observation supports the fact that this feedforward process may contribute to its cancer suppressor function. In individuals with neoplasia, USP11 expression is downregulated, which is associated with the expression level of PTEN and the location of FOXO within the nucleus. A feedforward loop responsible for this effect (i.e., stabilizing PTEN and promoting its cancer-suppressing function) comprises PTEN-PI3K-FOXO-USP11 [27].

In this role, USP11 is a key deubiquitinase for physiological PTEN which acts as an antagonist with respect to the PI3K/AKT signaling pathway [27]. Depletion through knockdown can substantiate its essential tumor suppressive function and its positive effects on PTEN, as described above. Decreasing the expression of PTEN might be a method that can inactivate PTEN without eradicating it. Polyubiquitination and protein integrity of PTEN in the cell cytoplasm and nucleus can be enhanced by USP11. In prostate cancer cells, USP11 has been shown to play a significant role in controlling PTEN titers and activity levels [27]. However, the expression of PTEN was not investigated in this study. We believe that these additional studies about PTEN will lead to a better understanding of the role of USP11.

The results obtained from the current study revealed that in comparison with samples exhibiting a higher USP11 grade, samples exhibiting a lower USP11 grade were associated with elevated PSA titers (*p* < 0.001), increased Gleason scores (*p* < 0.01), more advanced disease stage (*p* < 0.001), a greater degree of involvement of the seminal vesicles (*p* < 0.001) or lymph nodes (*p* < 0.001), and higher frequencies of positive surgical margins for tumors (*p* < 0.05) (Table 5, Table 6 and Table 7).

Cox proportional hazard analysis indicated that recurrence-free survival (both biochemical and clinical) and overall survival waere related to a lower USP11 grade. Similarly, the Kaplan Meier analysis, using the log-rank test, demonstrated that a lower USP11 grade on immunohistochemical staining was associated with both clinical and biochemical tumor recurrence.

## 4. Materials and Methods

### 4.1. Patients and Specimen Preparation

A total of 200 patients with a diagnosis of prostatic adenocarcinoma, confirmed both on histology and immunohistochemistry, were enrolled in this study. Radical prostatectomy (RP) was carried out between January 2002 and December 2012 at Soonchunhyang University Hospital in all cases. The patients’ medical charts were analyzed retrospectively, and data from before undergoing radical prostatectomy to 5 years after surgery (when data were available) were analyzed. A retrospective analysis was conducted in August 2022. Data of all patients were collected after processing so that basic patient information could not be identified. Tissue samples from those with prostatic adenocarcinoma were fixed in formalin and embedded in paraffin.

A representative pathological area was retrospectively highlighted on hematoxylin and eosin (H&E)-stained slides by two experienced pathologists. A retrospective review of pathological reports and additional clinical notes was also carried out to gather relevant medical data. International Union Against Cancer and World Health Organization/ International Society of Urological Pathology criteria were used to stage cancers and to allocate Gleason scores.

Serial PSA titers were monitored at different intervals (median, 132 months; range, 1–252 months) during the post-operative follow-up. Parameters included in the analysis were age, Gleason score, seminal vesicle invasion, lymph node involvement, surgical margin positivity, plasma PSA values, and disease stage. This study was conducted according to the principles of the Declaration of Helsinki. Approval was obtained from the local scientific ethics committees (Soonchunhyang University Seoul hospital, Bucheon Hospital, Cheonan Hospital, Gumi Hospital). We were granted a waiver for obtaining informed consent by the local scientific ethics committees for the following reasons: (1) the study did not conduct genetic testing of human origin; (2) the subject of the study was human-derived, and identification of the samples was not possible because the information of the subject was not provided or was not revealed to all research-related persons; (3) the information was coded and provided to the researcher without the subject identification record, or the personal information was processed and secured so that it was impossible to identify it by an impartial third party; (4) we had appropriate management guidelines and procedures for samples to prevent personal information exposure.

### 4.2. Construction of Tissue Microarrays

TMAs were generated from tissues fixed in formalin and embedded in paraffin blocks as noted above. An assiduous examination of H&E slides (*n* = 200) was performed under light microscopy in order to choose viable sections of malignancy that were deemed to be representative. Equivalent paraffin block sections were scored twice with a cylinder (diameter, 3 mm) and then relocated using a trephine device to a receiving block of paraffin (Superbiochips, Laboratories, Seoul, Republic of Korea). Control samples from the neighboring normal tissue (*n* = 8) were additionally prepared, with one block specimen being stained with H&E in order to verify the tissue type.

### 4.3. Purchase of Additional Tissues for Microarrays

Since there were insufficient numbers of patients with greater than stage 3 advanced malignancy in the study hospital, 90 human prostate cancer samples obtained following RP and 12 neighboring normal tissue specimens were procured from AccuMax (ISU ABXIS Co., Ltd., Seongnam, Republic of Korea). These comprised anonymized samples. Local pathologists verified that tissue H&E specimens were in fact malignant or benign as specified. These specimens were then prepared in an identical manner to those obtained from the local hospital.

### 4.4. Immunohistochemical Staining of Tissue Microarrays

TMAs were performed for human prostate cancer and control samples. Serial slices were sectioned (3 µm in thickness) from paraffin blocks containing TMA. Anti-USP11 (1:100 for immunofluorescence; KG403, Cosmo Bio, Tokyo, Japan), the primary antibody, was employed for immunohistochemical identification of USP11 expression. Mean staining intensity and proportion of cells showing positive staining were quantified using ImagePro V10 software (Media Cybernetics, Rockville, MD, USA). Non-malignant prostatic tissues were used as positive control. Each case comprised two sampled cores within the TMA.

The TMA paraffin slices were dewaxed with xylene, rehydrated with alcohol, added to Coplin jars containing tri-sodium citrate solution (0.01 M), and heated using a traditional pressure cooker for 3 min followed by 5 min of rinsing with cool running water. The samples were then rinsed with Tris-buffered saline (pH 7.4) prior to overnight incubation with a specific rabbit polyclonal antibody-USP11 (KG403, Cosmo Bio) at a 1:100 dilution. Tissue sections were then stained with biotinylated anti-rabbit immunoglobulins and peroxidase-labelled streptavidin (Dako, Carpinteria, CA, USA). Chromogen diaminobenzidine was the substrate utilized to enhance signals. Negative controls comprised samples that had a specific antibody omitted and pre-adsorbed. Particular attention was paid to assessing the expression of USP11 in well-preserved tissue cores. Based on histological scoring, the expression of staining was classified as follows by a pathologist, who was blinded to the patient tumor’s outcome data: grade 3—strong (+++), grade 2—moderate (++), grade 1—weak (+), and grade 0—negative (−). For statistical analysis, we re-classified USP11 expression into the following two grades: negative (negative) and positive (weak, moderate, and strong).

### 4.5. Definition of Survival Analysis

Biochemical recurrence was defined as a rise in the PSA titer ≥0.2 ng/mL on a minimum of two independent serial tests separated by a minimum of three months. Clinical recurrence was recognized as skeletal malignant deposits, lymph node involvement, or visceral spread as identified on radionuclide bone imaging or computed tomography of the abdomen, pelvis, and thorax.

Study participants were deemed at risk from the time of their RP until either recurrence or the date of the final PSA assessment. Participants lost to follow-up were censored at the final PSA or follow-up appointment date. The period from RP to death from any etiology was considered as the overall survival time.

### 4.6. Statistical Analysis

The following methods were used to examine the baseline characteristics of study subjects. Chi-square test or Fisher’s exact test was used for categorical data analysis. Student’s *t*-test or a Mann–Whitney U test was carried out to detect any differences between the two groups. Data were presented as *n* (%) and mean ± se or median (1st, 3rd quartile). Also, the McNemar test with continuity correction was used to confirm the relationship between USP11 immunohistochemical staining and prostate cancer.

Breslow estimator curves were generated for biochemical and clinical recurrence-free survival and overall survival. The log-rank test was utilized for data comparison. The expression of USP11 was analyzed statistically using Cox proportional hazard regression to determine any independent relationship with the aforementioned survival types. Subjects’ age at diagnosis, histological grade, pathological stage, PSA category, and lymph node involvement were taken into account. Univariable analyses were applied to the baseline study population variables and correlations between USP11 staining and recognized prognostic markers. Multivariate analysis was performed using the Akaike information criterion (AIC) for variable selection. As a result of checking the proportion assumption, all assumptions were satisfied. (*p* = 0.05 for biochemical recurrence, *p* = 0.154 for clinical recurrence and *p* = 0.078 for mortality) The Statistical Package for the Social Sciences (SPSS) version 26 was utilized for all data analyses. R program (version 3.6.3) was used with “survival” and “survminer” packages. *p*-values < 0.05 were deemed to indicate statistical significance.

## 5. Conclusions

Data of this study support the fact that USP11 immunohistochemistry can indicate the clinical outcomes in patients with prostate cancer. USP11 immunohistochemistry is also negatively correlated with the more traditionally used prognostic variables (i.e., PSA, Gleason score, disease stage, involvement of the seminal vesicles or lymph nodes, and a positive surgical margin). Therefore, USP11 staining is a valuable prognostic factor in patients with prostate cancer.

## Figures and Tables

**Table 1:** Clinicopathological properties and USP11 expression of patients with prostate cancer (*n* = 266).

Prognostic Factors	*n* (%)
Age	66 (61, 70)
PSA	
≤10	141 (53.82%)
10–20	69 (26.34%)
>20	52 (19.85%)
Gleason score	
≤6	41 (15.65%)
7–8	85 (32.44%)
8–10	52 (19.85%)
Pathological stage	
≤T2	112 (42.75%)
≥T3	150 (57.25%)
Seminal vesicle invasion	
Negative	146 (84.88%)
Positive	26 (15.12%)
Lymph node involvement	
Negative	165 (96.49%)
Positive	6 (3.51%)
Surgical margin *	
Negative	65 (55.23%)
Positive	77 (44.77%)
Expression of USP11 ^#^	115 (44.75%)
	142 (55.25%)

USP11 grade—0: negative, 1: positive. Age is expressed as median (1st, 3rd quartile). * the number of surgical margins could be evaluated in only 172 patients with prostate cancer at our hospital from 266 patients. ^#^ the amount of USP11 immunohistochemical expression could be evaluated in only 257 of 266 patients with prostate cancer.

**Table 2:** USP11 immunohistochemical staining of prostate cancer and adjacent non-neoplastic tissues for radical prostatectomy due to prostate cancer (*n* = 286).

UPS11	Adjacent Non-Neoplastic Tissues	Prostate Cancer	Total	*p*-Value
0	5	135	140	<0.05
1	15	131	146	

USP11 grade 0: negative, grade 1: positive.

**Table 3:** USP11 immunohistochemical staining of prostate cancer for radical prostatectomy due to prostate cancer.

	USP11		*p*-Value
	0 (*n* = 115)	1 (*n* = 142)	
Age	66 (62, 70)	66 (61, 70)	0.614
PSA			<0.001
≤10	44	92	
10–20	37	29	
>20	31	19	
Gleason score			<0.001
≤6	0	41	
7–8	4	80	
8–10	108	19	
Pathological stage			<0.001
≤T2	28	84	
≥T3	84	56	
Seminal vesicle invasion			<0.001
Negative	43	103	
Positive	17	9	
Lymph node involvement			0.1834
Negative	55	110	
Positive	4	2	
Surgical margin *			<0.05
Negative	26	69	
Positive	34	43	

USP11 grade 0: negative, grade 1: positive. Age is expressed as median (lower quadrant, upper quartile). * the number of surgical margins could be evaluated in only 172 patients with prostate cancer from 266 at our hospital, excluding purchased TMA.

**Table 4:** Biochemical recurrence-free survival time for USP11 expression (173 from 335 patients with prostate cancer could be evaluated for survival).

USP11	*n*	Event	Mean Survival Time (Estimated ± SE)	Log-Rank Test
Biochemical recurrence-free survival				
0	61	20	8.118 ± 0.666	0.000
1	112	10	11.991 ± 0.558	
Clinical recurrence-free survival				
0	61	9	10.111 ± 0.527	0.001
1	112	2	13.023 ± 0.350	
Overall survival				
0	61	12	10.014 ± 0.460	0.691
1	112	20	10.487 ± 0.552	

USP11 grade 0: negative, grade 1: positive.

**Table 5:** Cox proportional hazard modeling of USP11 after accounting for biochemical recurrence-free survival (*n* = 173).

	Univariable Analysis		Multivariable Analysis	
	HR (95% CI)	*p*-Value	HR (95% CI)	*p*-Value
Age	1.023 (0.962–1.087)	0.477		
USP11 grade				
0	Reference			
1	0.253 (0.115–0.556)	0.001	0.413 (0.180–0.951)	0.0338
PSA				
≤10	Reference			
10–20	1.648 (0.673–4.036)	0.274		
>20	2.364 (1.009–5.539	0.048		
Gleason score				
≤6	Reference			
7–8	1.545 (0.311–7.669)	0.595		
8–10	4.939 (1.158–21.070)	0.031		
Pathological stage				
≤T2	Reference			
≥T3	3.516 (1.672–7.392)	0.001		
Seminal vesicle invasion				
Negative	Reference			
Positive	4.444 (2.104–9.389)	<0.001	2.186 (0.963–4.965)	0.062
Lymph node involvement				
Negative	Reference			
Positive	5.863 (2.225–15.446)	<0.001	3.342 (1.209–9.236)	0.020
Surgical margin				
Negative	Reference			
Positive	5.861 (2.392–14.361)	<0.001	3.769 (1.454–9.772)	0.006

USP11 grade 0: negative, grade 1: positive.

**Table 6:** Cox proportional hazard modeling of USP11 after accounting for clinical recurrence-free survival (*n* = 173).

	Univariable Analysis		Multivariable Analysis	
	HR (95% CI)	*p*-Value	HR (95% CI)	*p*-Value
Age	1.095 (0.983–1.218)	0.099		
USP11 grade				
0	Reference			
1	0.075 (0.010–0.593)	0.014	0.241 (0.026–2.272)	0.214
PSA				
≤10	Reference			
10–20	1.632 (0.435–6.118)	0.468	1.325 (0.200–8.780)	0.770
>20	0.968 (0.186–5.039)	0.969	0.198 (0.023–1.716)	0.142
Pathological stage				
≤T2	Reference			
≥T3	3.008 (0.877–10.311)	0.080		
Seminal vesicle invasion				
Negative	Reference			
Positive	3.910 (1.083–14.121)	0.037	5.297 (0.835–33.606)	0.077
Lymph node involvement				
Negative	Reference			
Positive	13.802 (3.870–49.211)	<0.001	36.850 (3.131–433.667)	0.004
Surgical margin				
Negative	Reference			
Positive	3.341 (0.082–12.654)	0.076		

USP11 grade 0: negative, grade 1: positive.

**Table 7:** Cox proportional hazard modeling of USP11 after accounting for overall survival (*n* = 173).

	Univariable Analysis		Multivariable Analysis	
	HR (95% CI)	*p*-Value	HR (95% CI)	*p*-Value
Age	1.028 (0.968–1.091)	0.371		
USP11 grade				
0	Reference			
1	1.159 (0.559–2.400)	0.692	1.377 (0.651–2.991)	0.403
PSA				
≤10	Reference			
10–20	1.186 (0.509–2.765)	0.692		
>20	1.408 (0.605–3.273)	0.427		
Gleason score				
≤6	Reference			
7–8	0.945 (0.354–2.523)	0.909		
8–10	0.834 (0.318–2.186)	0.712		
Pathological stage				
≤T2	Reference			
≥T3	1.911 (0.946–3.859)	0.071		
Seminal vesicle invasion				
Negative	Reference			
Positive	2.441 (1.137–5.242)	0.022	2.631 (1.203–5.755)	0.015
Lymph node involvement				
Negative	Reference			
Positive	1.751 (0.530–5.791)	0.358		
Surgical margin				
Negative	Reference			
Positive	1.349 (0.664–2.740)		0.408	

USP11 grade 0: negative, grade 1: positive.

## Data Availability

The data presented in this study are available on request from the corresponding author. The data are not publicly available due to open restriction of local scientific committees.
